# Clinical Presentation of KMT2B-Related Dystonia: A Case Report

**DOI:** 10.7759/cureus.81454

**Published:** 2025-03-30

**Authors:** Elizabeth Onoprishvili, Luka Khelaia, Ana Bedoshvili, Nana Nino Tatishvili, Sofia Tatishvili

**Affiliations:** 1 Internal Medicine, David Tvildiani Medical University, Tbilisi, GEO; 2 Pediatric Medicine, David Tvildiani Medical University, Tbilisi, GEO; 3 Neurorehabilitation, Neurodevelopment Center, Tbilisi, GEO; 4 Pediatric Neurology, M. Iashvili Children's Central Hospital, Tbilisi, GEO; 5 Neurology, David Tvildiani Medical University, Tbilisi, GEO; 6 Physiotherapy, David Tvildiani Medical University, Tbilisi, GEO

**Keywords:** childhood-onset dystonia, dystonia, dystonia 28, dyt28, genetic diseases, kmt2b, kmt2b dystonia

## Abstract

This case presents an eight-year-old boy who visited the clinic with complaints of worsening gait and dystonic movements. The patient had asymmetric spasticity in all extremities, which was more pronounced on the right side. The diagnosis of *KMT2B*-related dystonia was made. *KMT2B*-related dystonia is a generalized dystonia of childhood-onset that typically begins in the lower limbs, gradually progressing upward and leading to generalized dystonia. The patient had prominent, involuntary hand movements, mainly on the right side, and difficulties with fine motor function. These symptoms severely impacted his ability to engage in routine tasks and daily activities. Currently, he is undergoing multidisciplinary rehabilitation treatment at the Neurodevelopment Center to enhance his functional abilities and participation and to improve his overall quality of life. Given its relative rarity in clinical practice, this case underscores the importance of early recognition and thorough documentation of *KMT2B*-related dystonia. By increasing awareness of this condition, healthcare providers can facilitate timely diagnosis and implement more effective treatment strategies. Early intervention can significantly improve outcomes and support children with this challenging disorder in leading fulfilling lives.

## Introduction

*KMT2B*-related dystonia is a rare childhood-onset movement disorder marked by pronounced muscle tone and gait abnormalities, which usually commence in the lower limbs and progress upward. It is associated with mutations in lysine-specific methyltransferase 2B (*KMT2B*) located on chromosome 19q13.12 [1]. *KMT2B*-related dystonia is most often inherited in an autosomal dominant manner, typically with heterozygous mutations. The median age of onset is five years (range: 1.5-29.0 years), with progression to generalized dystonia over a median period of two years (range: 0-10.5 years) [2].

Standard gross features include an elongated face, bulbous nasal tip, clinodactyly, hypothyroidism, precocious puberty, and ophthalmologic issues like refractive errors and nystagmus. Some skin and other systemic features, such as cyclical neutropenia, autoimmune hepatitis, or IgG deficiency, are also reported [2].

The dystonia becomes generalized over time (range one to nine years, mean 4.4 years) and often involves cervical (retrocollis and torticollis), oromandibular (facial dystonia and bulbar-oromandibular), and laryngeal (dysphonia and spasmodic laryngeal spasm) muscles [3]. The dystonia most commonly involves cervical, cranial, and laryngeal muscles. Patients typically exhibit involuntary muscle contractions and abnormal postures, significantly impacting their daily activities and quality of life. Additionally, communication difficulties are common due to articulation challenges and low speech volume. Bulbar dysfunction often leads to impaired swallowing. Intellectual disability (ID) or developmental delay (DD) is commonly reported [4]. Dysmorphic features such as microcephaly, short stature, and mild dysmorphic findings, including epicanthus, an elongated face, and a bulbous nasal tip, along with developmental delay, short stature, microcephaly, spasticity, hyperreflexia, eye movement abnormalities, dermatologic features, seizures, and psychiatric comorbidities, have also been described as phenotypic features in some cases [4,5].

The condition is often misdiagnosed due to its overlapping symptoms with other movement disorders. Given the limited number of case reports on this condition, we aim to contribute to the existing body of knowledge and provide new insights. This report seeks to illustrate this patient's clinical presentation, diagnostic challenges, and management strategies, thereby contributing to understanding such rare cases.

## Case presentation

The patient is an eight-year-old boy who presented with involuntary movements and gait instability to the Neurodevelopment Center in June of 2023 in Tbilisi, Georgia. The patient was delivered via cesarean section at 33 weeks of gestation to a multiparous mother of two (nonconsanguineous parents). The patient's mother had a complicated first pregnancy that resulted in neonatal death. Her second pregnancy was uneventful, and the child from that pregnancy, a now 10-year-old male, is healthy. Our patient, born from her third pregnancy, did not have immediate respiratory difficulties such as asphyxia. However, soon after birth, he developed signs of sepsis, requiring admission to the neonatal intensive care unit, where he received antibiotic therapy. He has one older sibling, the previously mentioned 10-year-old healthy male. The family history was unremarkable. Early neuromotor development was normal; he could sit independently at six months and communicate and walk independently at 12 months.

**Figure 1:**
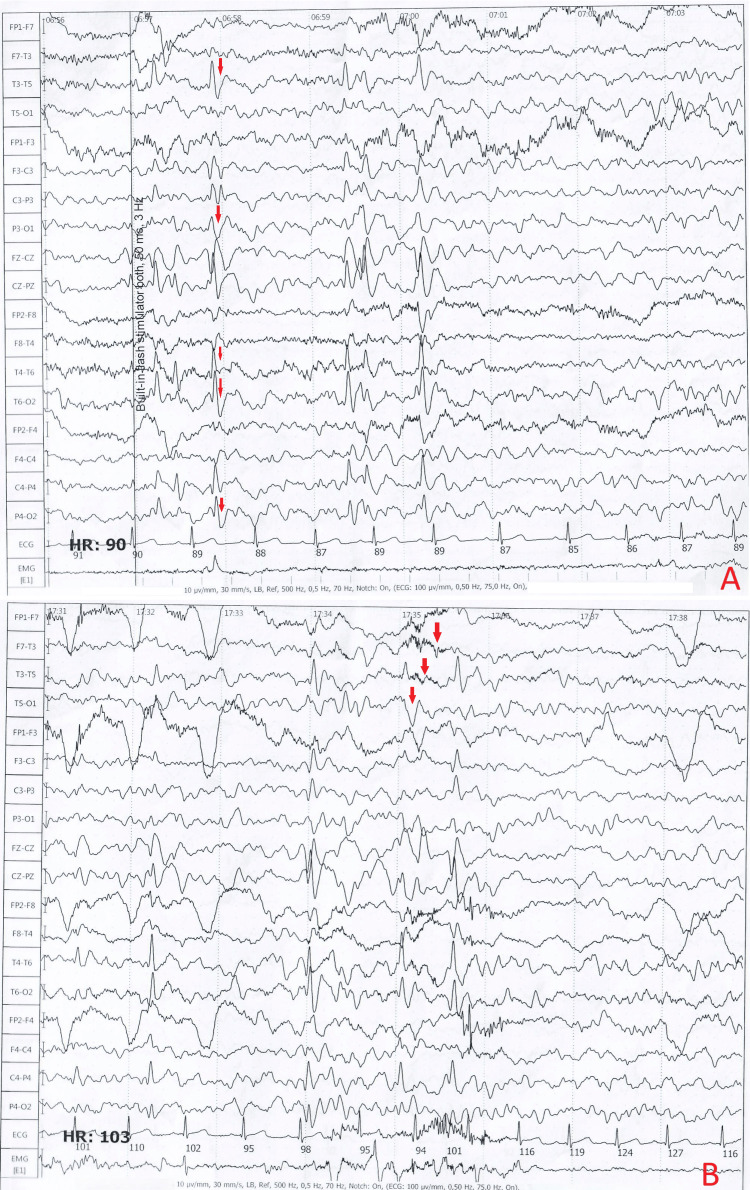
Electroencephalographic patterns (left temporal epileptiform activity and photosensitive response in the occipital region) A: epileptiform discharges localized to the left temporal region, characterized by spike-and-wave complexes, high-amplitude rhythmic activity, and polymorphic sharp waves (indicated by red arrows); B: demonstrates photosensitive epilepsy, with spike-and-wave and polyspike discharges (indicated by red arrows) localized to the occipital and posterior temporal regions, markedly enhanced by photic stimulation.

**Figure 2:**
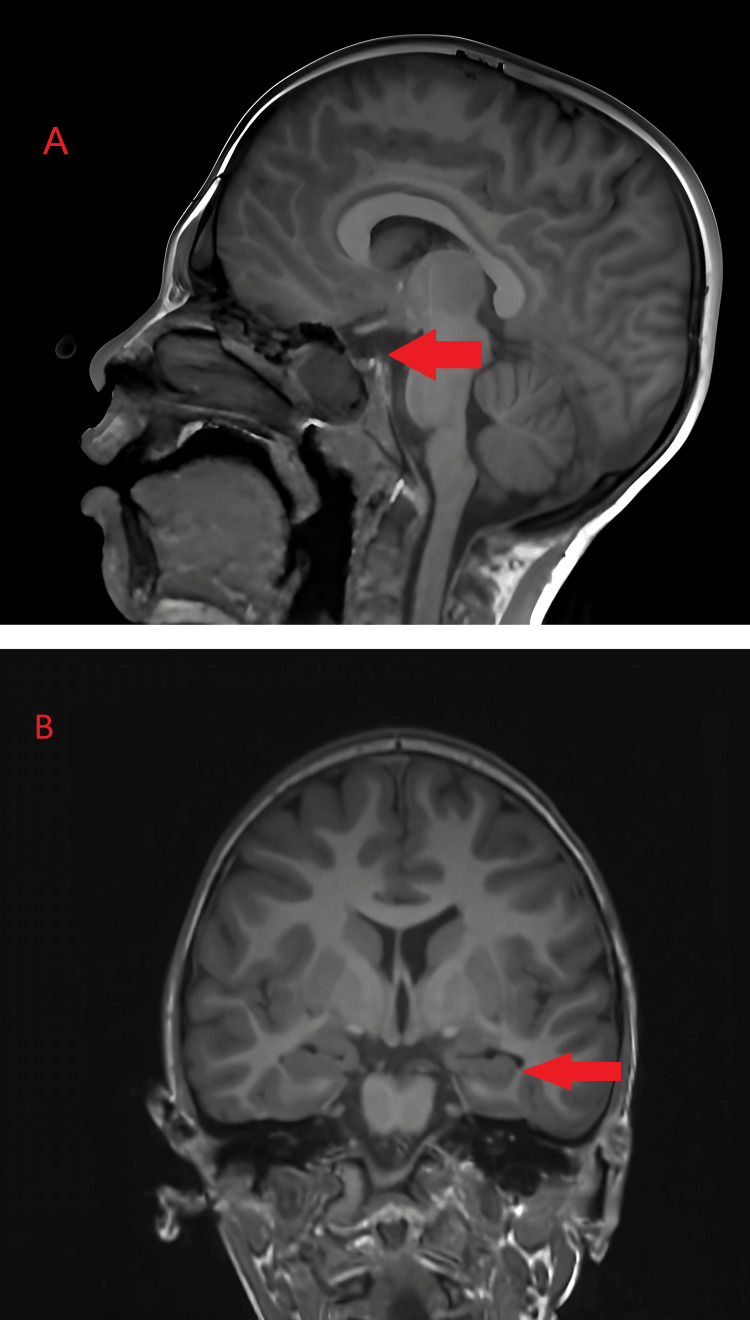
Magnetic resonance imaging (MRI) demonstrating structural abnormalities in the brain T2-weighted magnetic resonance imaging (MRI) showing (A) sagittal section showing a hypointense region in the posterior fossa (red arrow), suggestive of hypoplasia of the pituitary; (B) coronal section showing hippocampal gliosis marked by increased signal intensity in the hippocampal region (red arrow).

At the age of four years, the parents noted involuntary mouth movements during sleep, during which they had difficulty waking him. The patient was admitted to the hospital, where diagnostic evaluation, including clinical examination, electroencephalogram (Figure 1), and MRI (Figure 2), and antiepileptic treatment was initiated (Table 1). Involuntary movements of the right extremities were noticed one year later, which was considered an epileptic seizure, and valproic acid was prescribed. The patient's condition worsened, and carbamazepine was added, which caused side effects such as speech difficulties and increased involuntary movements; therefore, all medications were withdrawn. 

**Table 1:** Tests/Assessments performed GRCh37: Genome Reference Consortium Human Build 37; rCRS: Revised Cambridge Reference Sequence (of the mitochondrial genome); RefSeq: NCBI Reference Sequence database; SNV: single nucleotide variant; ACMG: American College of Medical Genetics and Genomics; AMP: Association for Molecular Pathology; MRI: magnetic resonance imaging; EEG: electroencephalogram; IQ: intelligence quotient; EVIDENCE software (3billion, Inc., Seoul, South Korea, http://3billion.io/)

Test/Assessment	Findings
Genetic Test	Whole-exome sequencing (WES) identified a heterozygous variant of uncertain significance (VUS) in the *KMT2B* gene (Genomic position: 19-36229049-G-A, DNA: NM_014727.3: c.7829G>A, Protein: NP_055542.1: p. Arg2610His), associated with autosomal dominant childhood-onset dystonia. Validation was not performed as the variant was considered high-quality. Methods: High-quality exome sequencing of a blood sample specimen yielded extensive coverage (144.82x mean depth over 99.3% of RefSeq coding regions, with 99.00% ≥20x). A total of 66,952 SNVs and 11,163 indels were identified from 9,994,059,014 aligned bases (GRCh37/rCRS). The variant interpretation was performed using EVIDENCE software following ACMG/AMP guidelines, considering patient phenotype and history. Only clinically significant and relevant variants will be reported, with low-quality variants confirmed by Sanger sequencing. Resources: Online Mendelian Inheritance in Man, Genome Aggregation Database (gnomAD), National Center for Biotechnology (ClinVar), Human Genome Variation Society (HGVS), the Human Gene Mutation Database (HGMD) Professional.
EEG (Figure 1)	Revealing focal interictal epileptiform activity.
Brain MRI (Figure 2)	Hippocampal gliosis and hypoplasia of the pituitary gland.
Intellectual Assessment	Wechsler Intelligence Scale for Children (WISC-II) - Total IQ = 50 (Verbal IQ = 55, Nonverbal IQ = 50), leading to a diagnosis of mild intellectual disability.
Oral Motor Measurement Scale (OMMS)	Trunk: The patient avoids chewing during meals and attempts to swallow quickly. His speech is flat, low, and rapid, with difficulty maintaining a coherent narrative.
Gross Motor Function Classification (GMFCS)	Classification (GMFCS)Level II. The patient uses a thoracic-lumbar orthosis, as prescribed by an orthopedist.
X-ray of Pelvis and Femur	Revealed congenital dysplasia of the right hip joint.

Cranial nerve assessment showed no abnormalities. The patient has a slightly hoarse voice with mildly impaired articulation, increased muscle tone, and brisk deep tendon reflexes in all extremities, more on the right side. No clonus and pathological reflexes are present. There are notable involuntary and dystonic movements, which are more prominent in the fingers and are brought on by emotional status. Coordination tests are abnormal. Motor skill deficits impact the patient's ability to perform daily tasks. He struggles with gait and has difficulty walking in a straight line. He fails a 4.5m run-and-return test because he cannot initiate the running motion. He also cannot initiate jumping, either in place or forward, showing affected gross motor skills. He fastens his shoes with hook-and-loop closures (Velcro) independently but cannot tie shoelaces. He can draw but experiences writing challenges due to impaired fine motor control in his right hand, which affects his pen grip. Given the unusual presentation of symptoms and the lack of response to medications, alternative diagnoses were considered, including cerebral palsy. However, due to the progressive nature of the disease, further differential diagnoses were explored, prompting the decision to pursue genetic testing. The results revealed a heterozygous variant of uncertain significance (VUS) in the *KMT2B* gene, and upon correlating this with the clinical presentation, *KMT2B* dystonia was strongly suggested as the diagnosis (Table 1).

Deep brain stimulation (DBS) is considered to be one treatment option for this condition. Meta-analyses state that DBS of the globus pallidus internus has better outcomes in patients with more severe dystonia. This was why the treatment team did not proceed with DBS at this stage. The patient receives multidisciplinary rehabilitation treatment, including physical, occupational, and speech and language therapy.

## Discussion

This case of an eight-year-old boy with *KMT2B*-related dystonia illustrates the diagnostic and therapeutic challenges of managing a rare, childhood-onset genetic disorder. The case demonstrates the complexities of diagnosing and managing a condition with diverse and progressive clinical manifestations.

In this case, the patient began showing early signs of the disease at age four, progressing to involuntary right-side movements by age five. The constellation of asymmetric hypertonia and involuntary movements led to the initiation of valproate and, one year later, carbamazepine, which accentuated or caused needless side effects like the new emerging speech difficulty. This emphasizes the importance of a thorough diagnostic approach, particularly the role of genetic testing, which ultimately identified a variant of uncertain significance in the *KMT2B* gene.

As information provided in existing literature, this patient exhibits several hallmark features of *KMT2B*-related dystonia, including motor impairments, intellectual disability, and developmental delay [6]. His clinical course also aligns with reports of progressive symptom worsening and the involvement of various muscle groups, impacting his ability to perform daily tasks. The MRI findings of hippocampal gliosis and pituitary hypoplasia are less commonly reported in *KMT2B*-related dystonia. Literature states that the most frequently seen abnormality is the hypointensity of bilateral globus pallidus; however, our patient did not demonstrate it [4,7]. They may represent comorbidities rather than direct effects of the gene variant, adding complexity to the case and potentially affecting cognitive function and endocrine regulation.

The patient receives physical, occupational, and speech therapies to improve his quality of life. No pharmacological treatment has been initiated for this patient because his symptoms are moderate. As stated, bilateral globus pallidus internus deep brain stimulation (DBS) was considered, but as the patient's condition was not severe, managing physicians decided against advising it. If the condition progresses, the physicians will begin discussing the potential implementation of this treatment. This decision is based on information gathered from meta-analyses suggesting that the outcome of globus pallidus internus DBS is better in patients with more severe dystonia [8].

Although the patient's symptoms largely align with the typical manifestations described in existing literature, some unique characteristics are notable. Specifically, the hypertonia presented asymmetrically, with greater severity in the distal limbs rather than the more typical truncal or axial distribution [4].

One significant challenge in managing and diagnosing rare genetic diseases is distinguishing these conditions from more common diseases with overlapping symptoms. Due to familiarity and prevalence, clinicians may often initially consider a more common disorder, like cerebral palsy, especially when specific symptoms align closely with those of rare genetic conditions. This can lead to misdiagnosis and delays in targeted treatment, which is particularly impactful in the case of syndromes with subtle or complex presentations.

For instance, *KMT2B*-related dystonia shares several overlapping symptoms with cerebral palsy, which can complicate the diagnostic process. Both conditions may present with motor abnormalities, such as spasticity, dystonia (abnormal muscle tone and movement), and difficulties with coordination [4,9]. Additionally, developmental delays, speech and language challenges, and abnormalities in gait are standard features in both cerebral palsy and *KMT2B* dystonia. Other differential diagnoses that could be considered are tics, particularly “dystonic” tics, essential, and myoclonus, with its characteristic sudden, jerky movements that can resemble dystonia [10].

However, distinguishing features do exist and can guide physicians toward a more accurate diagnosis. In *KMT2B*-related dystonia, symptoms often worsen over time, whereas cerebral palsy is usually a non-progressive condition. Genetic testing becomes invaluable here, as identifying a mutation in the *KMT2B* gene can confirm the diagnosis, distinguishing it from cerebral palsy and guiding more precise treatment options. A novel test could also more effectively identify *KMT2B* dystonia by measuring histone H3K4 trimethylation in oral mucosa [11,12]. However, further evidence and testing are required.

For physicians involved in diagnosing and managing this disease in the future, we strongly recommend initiating genetic testing as soon as there is evidence of clinical progression. By confirming a genetic basis early on, clinicians can avoid unnecessary treatments that might otherwise target unrelated differential diagnoses. Progression also helps exclude cerebral palsy from a diagnosis. This approach not only helps to prevent potential side effects from unwarranted treatments but also reduces healthcare costs and minimizes patient burden, ultimately leading to more effective care and improved outcomes.

## Conclusions

This case report illustrates the complex clinical presentation, diagnostic challenges, and management strategies associated with *KMT2B*-related dystonia. It also shows that this rare genetic disorder can easily be mistaken for more common movement disorders like cerebral palsy. The patient’s unique features, including asymmetric hypertonia, involuntary dystonic movements, and atypical neuroimaging findings such as hippocampal gliosis and pituitary hypoplasia, underscore the disorder's heterogeneity. Early and targeted genetic testing is critical in distinguishing *KMT2B*-related dystonia from other conditions, especially when symptoms exhibit a progressive trajectory. Genetic testing revealing a variant of uncertain significance in the *KMT2B* gene proved pivotal in establishing the diagnosis. This suggests that even variants labeled as "uncertain" can hold diagnostic value when supported by strong clinical evidence.

The current multidisciplinary rehabilitation approach comprising physical, occupational, and speech therapies has been instrumental in enhancing the patient’s functional abilities and quality of life. At the same time, deep brain stimulation remains a consideration should the condition progress. By addressing both the distinctive clinical features and the challenges in diagnosis and treatment, this report contributes valuable insights into the spectrum of *KMT2B*-related dystonia. It reinforces the need for early, targeted interventions.

Given the rarity of *KMT2B*-related dystonia, existing evidence remains limited. Thus, each case report, including this one, provides valuable insights that contribute to a more comprehensive understanding of the disorder, aiding healthcare providers in recognizing its unique presentation. Continued research and case documentation are essential to refining diagnostic and management strategies, ultimately leading to better outcomes for children facing this challenging condition.
